# Supplementary material for: Impact on Patient Management of a Novel Host Response Test for Distinguishing Bacterial and Viral Infections: Real World Evidence from the Urgent Care Setting
Source: Biomedicines. 2023 May 22;11(5):1498. doi: 10.3390/biomedicines11051498 (PMC10216409; doi:10.3390/biomedicines11051498)
Supplement: Supplementary file 1 [file biomedicines-11-01498-s001.zip › biomedicines-2359133-supplementary.pdf]

Supplementary Figures

Figure S1 Translation of the questionnaire. Physicians were required to answer questions 1–3 prior to receiving the BV result and questions 4–5 after receiving the results.

Note: this is a translated version of the questionnaire

Maccabi Pilot Questionnaire:

Q1: In your opinion, what is the suspected infection etiology of the patient?

- High likelihood of viral infection
- Moderate likelihood of viral infection
- Undecided
- Moderate likelihood of bacterial infection
- High likelihood of bacterial infection

Q2: Do you intend to treat with antibiotics?

- Very high likelihood
- High likelihood
- Uncertain
- Low likelihood
- Very low likelihood

Q3: What other tests were ordered for this patient?

- CBC
- Chemistry
- Urinalysis
- Urine culture
- X ray
- Other

Q4: How did the MeMed BV result impact your decision-making process regarding this patient's management?

- Changed
- Supported
- Other (please specify)

Q5: What additional steps followed the BV test result?

- Culture
- Imaging
- ED referral
- Other:

Figure S2 Schematic of the study design

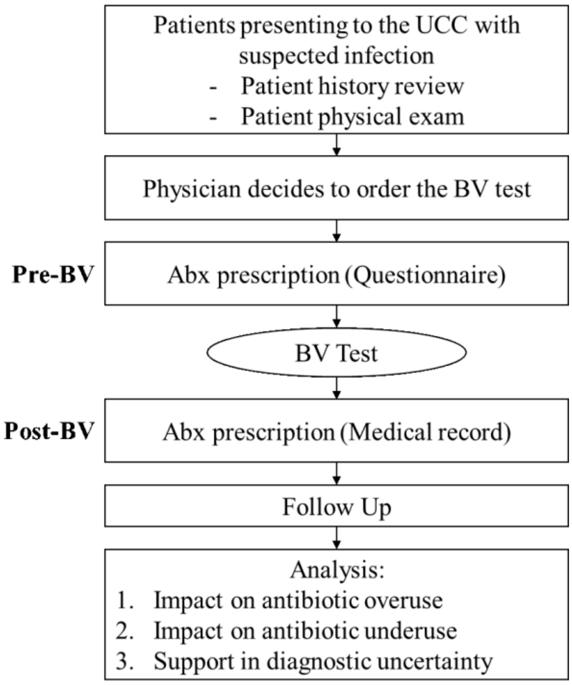

Figure S3 Only for cases with equivocal BV results (n=21): Physician's original intent regarding antibiotic prescriptions (intent), the BV result and whether antibiotics were prescribed according to the medical records (practice). Practice is reported as the percentage of bacterial/viral cases for whom antibiotics were prescribed (gray) or not prescribed (white), and the number of cases is in brackets.

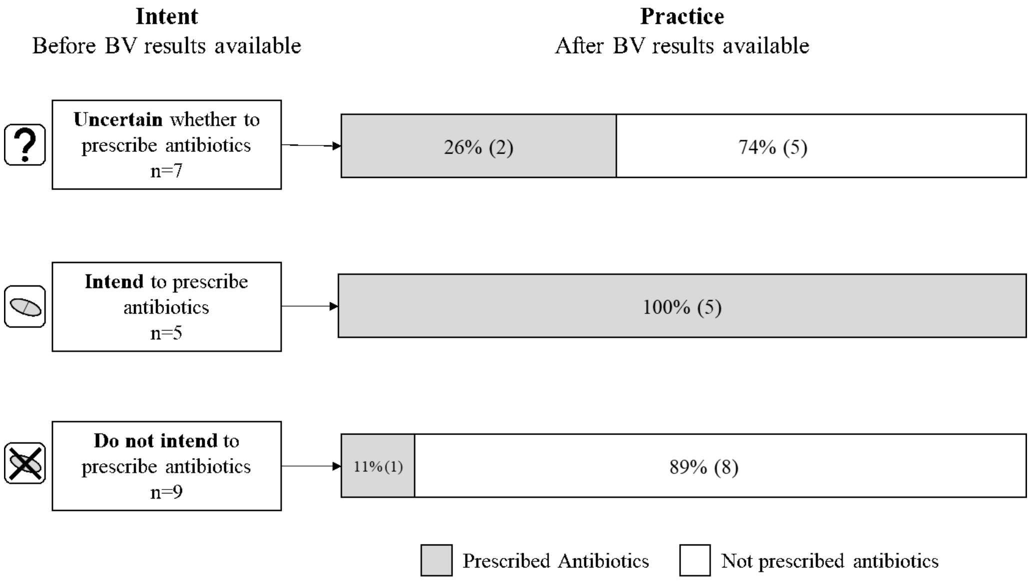

Supplementary Tables

Table S1- **Characteristics of the study population broken down based on intention to prescribe antibiotics**

		**Intention to treat**, N = 39	**No intention to treat**, N = 54	**Uncertain**, N = 38
Age	**All**	11 (1-46)	3 (1-39)	15 (3-36)
	**Children**	1.5 (1.0-3.0)	1.2 (0.6-1.9)	2.7 (1.8-7.1)
	**Adults**	44 (25-52)	38 (29-46)	41 (31-47)
	**Sub-cohort: Elderly; ≥65**	74 (74-80)	77 (75-80)	NA
Sex	**Female**	18 (46%)	25 (46%)	24 (63%)
Acute illness	**Days from symptoms onset (Median, IQR)**	2.00 (1.25-3.00)	2.50 (1.00-4.00)	2.00 (1.00-4.00)
	**Prescribed antibiotics at UCC**	30 (77%)	11 (20%)	15 (39%)

		**Intention to treat**, N = 39	**No intention to treat**, N = 54	**Uncertain**, N = 38
Discharge Diagnosis				
	Fever	9 (23%)	18 (33%)	10 (26%)
	Viral Infection	4 (10%)	14 (26%)	5 (13%)
	Pneumonia	7 (18%)	2 (3.7%)	4 (11%)
	Urinary Tract Infection / Pyelonephritis	6 (15%)	2 (3.7%)	4 (11%)
	Upper Respiratory Infection	3 (7.7%)	6 (11%)	2 (5.3%)
	Tonsillitis / Pharyngitis	4 (10%)	3 (5.6%)	3 (7.9%)
	Other	6 (15%)	9 (17%)	10 (26%)
Follow up (7 days)	**Hospitalized**	1 (2.6%)	4 (7.4%)	5 (13%)

Table S2 Case descriptions of patients for whom physicians indicated diagnostic uncertainty and acted in accordance with a viral BV result, that were prescribed antibiotics within 7 days of UCC discharge (n=3).

									Follow Up				
Age	Sex	Comorbidity	Days from symptom onset	CRP (mg/dL)	BV Score	ANC (10^9/L)	CXR	Microbiology	ED Referral	Discharge Diagnosis	Re-visit to GP/ED	Abx in re-visit	Description/Notes
35	F		0	---	25	---	No	CMV – Pos.	No	URI	Yes-GP	Yes	A 35-year-old female, presented with sore throat and no fever nor cough. Exudates found in physical examination. Normal WBC, CRP not measured. Diagnosed with URI and discharged without antibiotics. Visited GP within 7 days and prescribed antibiotics.
2	M		7	7.3	21	7.1	No	Blood culture – Neg.<br>EBV – Neg.	No	Fever	Yes-ED	Yes	A 2-year-old male presented with 7 day of fever and without other symptoms. Visited the GP 3 times prior to UCC visit (within the last 7 days). Physical examination without significant findings. Slightly elevated CRP. Discharged with a diagnosis of fever and not prescribed antibiotics. The patient presented to the ED within 7 days of UCC discharge and was hospitalized with pneumonia. Hospitalized for 3 days and treated with antibiotics.
42	F		2	3.4	1	3.13	Normal	Urine culture – Neg.<br>Covid-19 – Neg.	No	Fever	Yes-GP	Yes	A 42-year-old female presented with 2 days of fever, sore throat, weakness and nausea. Already under antibiotic treatment. No significant findings in physical exam. Normal CRP and WBC. Urinalysis with leukocyturia and erythrocyturia, without nitrites. Discharged with a diagnosis of fever and not prescribed antibiotics. Visited GP within 7 days and prescribed antibiotics.

Table S3 Case descriptions of patients for whom physicians initially intended to prescribe antibiotics, received a viral BV result and eventually did not prescribe antibiotics (n=9)

											Follow up		
**Age**	**Sex**	**Comorbidity**	**Days from symptom onset**	**CRP (mg/dL)**	**BV Score**	**ANC (10^9/L)**	**ED Referral**	**CXR**	**Microbiology**	**Discharge Diagnosis**	**Re-visit to GP/ED**	**Abx in re-visit**	**Description/Notes**
60	F		**0**	<0.5	1	3.6	No	No	Urinalysis – Neg. Urine culture – Neg.	Flank pain	No	No	A 60-year-old female presented with chills and nausea. Flank pain noted on physical examination.. Diagnosed with flank pain and discharge without abx treatment.
1.1	F		**1**	0.7	1	8.8	No	Nor mal	Blood culture – Neg.	Viral inf.	Yes – GP	No	A 1.1-year-old female. Was seen by GP one day prior to UCC visit, diagnosed with fever and treated with abx. Presented to the UCC with 1 days of fever, rhinorrhea and vomiting. Physical examination without significant finding. Slightly elevated WBC. Diagnosed with viral infection and discharge without abx treatment.
37	F		**4**	<0.5	1	3.29	No	Nor mal	Throat culture – Neg.	URI	No	No	A 37-year-old female presented with 4 days of fever, cough, rhinorrhea and sore throat. Physical examination without significant finding. Diagnosed with URI and discharge without abx treatment.
1.4	M	Cardiovascular Disease	**9**	ND	2	6.63	No	Nor mal	---	Viral inf.	Yes – GP	No	A 1.4-year-old male presented with 9 days of fever, cough and rhinorrhea. Visited GP in the previous week and diagnosed with otitis media. On physical examination, red throat and exudate. Diagnosed with viral infection and discharge without abx treatment.
47	F		**4**	2.7	2	1.57	No	No	Urine culture – Neg.	Viral inf.	Yes – GP	No	A 47-year-old female presented with 4 days of fever, chills, weakness and sore throat. Physical examination without significant finding. Diagnosed with viral infection and discharge without abx treatment. On GP visit in the coming week, diagnosed with post covid vaccine symptoms.
45	F		**3**	ND	5	7.42	No	Nor mal	---	Viral inf.	No	No	A 45-year-old female presented with 3 days of fever, cough and vomiting. Physical examination without significant finding. Slightly elevated WBC. Diagnosed with viral infection and discharge without abx treatment.
17.3	M		**2**	ND	7	6.26	No	No	Throat culture – Neg.	Tonsillitis	No	No	A 17.3-year-old male presented with 2 days of fever and sore throat. Exudate noted on physical examination. Diagnosed with tonsillitis and discharge without abx treatment.
66	F	Diabetes	**10**	<0.5	12	6.2	No	Nor mal	---	Fever	Yes – GP	No	A 66-year-old female presented with 10 days of fever, conjunctivitis, cough and sore throat. Visited ED in the previous week and diagnosed with URI, no abx prescribed. Physical examination without significant finding. Diagnosed with fever and discharge without abx treatment.
25	F		**0**	ND	15	6.91	No	No	Throat culture – Pos. (*Strep A*)	URI	Yes – GP	Yes	A 25-year-old female presented with sore throat. Visited GP in the previous week and diagnosed with URI. Exudate noted on physical examination. Diagnosed with URI and discharged without abx treatment. On re-visit to GP, throat culture was positive and abx was prescribed.

Table S4 Case descriptions of patients for whom physicians initially intended not to prescribe antibiotics, received a bacterial BV result and eventually prescribed antibiotics (n=8)

											Follow Up		
**Age**	**Sex**	**Comorbidity**	**Days from symptom onset**	**CRP (mg/dL)**	**BV Score**	**ANC (10^9/L)**	**CXR**	**Microbiology**	**ED Referral**	**Discharge Diagnosis**	**Re-visit to GP/ED**	**Abx in re-visit**	**Description/Notes**
86	F	Cardiovascular Disease ; Diabetes	4	23.1	99	9.1	RUL inf.	---	Yes	Pneumonia	No	No	A 86-year-old female presented with 4 days of fever and myalgia. Physical examination without significant finding. Slightly elevated WBC. Treated with abx and referred to the ED. Hospitalized for 6 days with pneumonia.
75	M	Diabetes	2	ND	98	14.11	No	Urine culture – Neg.	No	Pyelonephrit is	Yes	Yes	A 75-year-old male presented with 2 days of fever and urinary complaints. Visited GP in the previous week and diagnosed with fever. Physical examination without significant finding. Elevated WBC. Diagnosed with pyelonephritis and treated with abx.
2.1	F		2	12.9	97	5.9	Norm al	Urine culture – Neg.	No	Fever	Yes	Yes	A 2.1-year-old female presented with 2 days of fever and ears pain. Visited GP in the previous week and diagnosed with otitis externa. Physical examination without significant finding. Negative urinalysis. Diagnosed with fever and discharged with abx treatment.
45	F		0	12.7	97	6.8	Norm al	Covid-19 – Neg.	N0	URI	No	No	A 45-year-old female presented with rhinorrhea, weakness and headaches. Physical examination without significant finding. Diagnosed with URI and discharged with abx treatment.
29	M	Cardiovascular Disease	3	20.8	97	8.6	Norm al	---	Yes	Fever	Yes	No	A 29-year-old male presented with 3 days of fever, myalgia, chills and weakness. Visited the ED in the previous week. Physical examination without significant finding. Diagnosed with fever and referred to the ED. Hospitalized for 3 days with pneumonia and pericarditis, treated with abx.
0.6	M		4	ND	88	10.09	Norm al	---	No	Tonsillitis	No	No	A 0.6-year-old male presented with 4 days of fever. Visited GP in the previous week and diagnosed with fever. On physical examination, red throat and exudate. Slightly elevated WBC. Diagnosed with tonsillitis and treated with abx.
55	F	COPD	0	10.9	85	10.79	No	---	No	Acute bronchitis	Yes	Yes	A 55-year-old female presented with cough and rhinorrhea. Was seen by GP 3-days prior to UCC visit, diagnosed with viral infection and treated with abx. Physical examination without significant finding. Elevated WBC. Diagnosed with acute bronchitis and discharged with abx treatment.
58.9	F		1	5.7	73	9.09	Inf.	---	No	Pneumonia	No	No	A 58.9-year-old female presented with 1 day of fever, cough and sore throat. Crepitations noted on auscultation to the lungs. Slightly elevated WBC and infiltrate on chest x-ray. Diagnosed with pneumonia and discharged with abx treatment.

Table S5 Case descriptions of all elderly patients (over 65 years old, n=11). Highlighted cases indicate the BV result caused a change in patient management according to the physician.

Age	Sex	Comorbidity	Days from symptom onset	CRP (mg/dL)	BV Score	ANC (10^9/L)	CXR	Microbiology	ED Referral	Discharge Diagnosis	Follow Up: Re-visit to GP/ED	Follow Up: Abx in re-visit	Description/Notes
74	M	Cardiovascular Disease; Obstructive Lung Disease	2	35	100	27.4	LLL Inf.	Covid-19 – Neg.	Yes	Pneumonia	Yes	Yes	A 74-year-old male presented with 2 days of fever, cough, rhinorrhea, chills and weakness. Looks weak, and crepitations and decreased breath sounds noted on auscultation to the lungs. Elevated WBC. Diagnosed with pneumonia, treated with abx and referred to the ED. Hospitalized for 5 days with pneumonia and abx treatment.
86	F	Cardiovascular Disease; Diabetes	4	23.1	99	9.1	RUL Inf.	---	Yes	Pneumonia	No	No	A 86-year-old female presented with 4 days of fever and myalgia. Physical examination without significant finding. Slightly elevated WBC. Treated with abx and referred to the ED. Hospitalized for 6 days with pneumonia.
75	M	Diabetes	2	NA	98	14.11	No	Urine culture – Neg.	No	Pyelonephritis	Yes	Yes	A 75-year-old male presented with 2 days of fever and urinary complaints. Visited GP in the previous week and diagnosed with fever. Physical examination without significant finding. Elevated WBC, negative urine culture. Diagnosed with pyelonephritis and treated with abx.
84	M	Cardiovascular Disease	2	15.7	98	7.81	Normal	Covid-19 – Neg.	No	Fever	No	No	A 84-year-old male presented with 2 days of fever, cough, rhinorrhea and earache. Visited GP in the previous week and diagnosed with cough. On physical examination, impression of left ear otitis externa. Diagnosed with fever and discharged with abx treatment.
74	M	Obstructive Lung Disease	1	1.8	86	16.49	No	Urine culture – Pos. (*E. coli*)	No	UTI	Yes	No	A 74-year-old male presented with 1 day of fever, chills, vomiting and urinary complaints. Looks unwell, but the rest of the physical examination without any other significant finding. Elevated WBC. Diagnosed with UTI and discharged with abx treatment.
71	F	Hemato-oncological disease	3	11.4	37	1.88	Normal	Blood culture – Neg. Urine culture – Neg.	No	Fever	Yes	No	A 71-year-old female presented with 3 days of fever and weakness. Red throat noted on physical examination. Diagnosed with fever and discharged with abx treatment.
81	F	Diabetes	2	4	30	6.6	No	Urine culture – Neg.	No	UTI	Yes	Yes	A 81-year-old female presented with 2 days of fever, myalgia and weakness. Physical examination without significant finding. Urinalysis with leukocyturia and erythrocyturia, without nitrites. Diagnosed with UTI and discharged with abx treatment.
76	F	Diabetes	0	3.6	26	5.38	No	---	No	Cough	Yes	No	A 76-year-old female presented with cough and weakness. Looks pale, but the rest of the physical examination without any other significant finding. Urinalysis with leukocyturia and erythrocyturia, without nitrites. Diagnosed with cough and discharged without abx treatment.
80	M		2	5.1	23	4.72	No	Urine culture – Pos. (*E. coli*)	No	UTI	Yes	Yes	A 80-year-old male presented with 2 days of fever, chills and urinary complaints (hematuria). Physical examination without significant finding. Diagnosed with UTI and discharged with abx treatment.
66	F	Diabetes	10	<0.5	12	6.2	Normal	---	No	Fever	Yes	No	A 66-year-old female presented with 10 days of fever, conjunctivitis, cough and sore throat. Visited ED in the previous week and diagnosed with URI, no abx prescribed. Physical examination without significant finding. Normal chest x-ray. Diagnosed with fever and discharge without abx treatment.
77	M	Cardiovascular Disease	6	4.7	5	4.34	Normal	---	No	Viral infection	Yes	No	A 77-year-old male presented with 6 days of fever, headaches and urinary complaints. Was seen by GP 1-day prior to UCC visit, diagnosed with UTI and treated with abx. Physical examination without significant finding. Normal chest x-ray. Diagnosed with viral infection and discharged without abx treatment. After UCC discharge, hospitalized for 3 days with pneumonia and treated with abx.
71	F	Obstructive Lung Disease	6	<0.5	4	4	Normal	Urine culture – Neg.	No	Viral infection	Yes	No	A 71-year-old female presented with 6 days of fever, cough and rhinorrhea. Was seen by GP 3-days prior to UCC visit, diagnosed with bronchitis and treated with abx. Physical examination without significant finding. Diagnosed with viral infection and discharged without abx treatment.
